# A novel p.A191D matrilin-3 variant in a Vietnamese family with multiple epiphyseal dysplasia: a case report

**DOI:** 10.1186/s12891-020-03222-4

**Published:** 2020-04-07

**Authors:** Thuong Thi Ho, Linh Huyen Tran, Lan Thu Hoang, Phuong Kim Thi Doan, Trang Thi Nguyen, Trang Hong Nguyen, Hoai Thu Tran, Ha Hoang, Ha Hoang Chu, Anh Lan Thi Luong

**Affiliations:** 1 National Key Laboratory of Gene Technology, Institute of Biotechnology (IBT), Vietnam Academy of Science and Technology (VAST), 18 Hoang Quoc Viet, Cau Giay, Ha Noi, Viet Nam; 2 Graduate University of Science and Technology, Vietnam Academy of Science and Technology, 18 Hoang Quoc Viet, Cau Giay, Ha Noi, Viet Nam; 3 Department of Biology & Medical Genetic, Hanoi Medical University, 1 Ton That Tung, Dong Da, Ha Noi, Viet Nam; 4 Genetic Counseling Center, Hanoi Medical University Hospital, 1 Ton That Tung, Dong Da, Ha Noi, Viet Nam

**Keywords:** Multiple epiphyseal dysplasia, *MATN3*, Vietnamese case, Heterozygous, de novo missense variant, P.A191D matrilin-3

## Abstract

**Background:**

Multiple epiphyseal dysplasia (MED) is a common skeletal dysplasia that is characterized by variable degrees of epiphyseal abnormality primarily involving the hip and knee joints. Mutations in a gene encoding matrilin-3 (*MATN3*) have been reported as disease causing of autosomal dominant MED. The current study identified a novel c.572 C > A variant (p.A191D) in exon 2 of *MATN3* in a Vietnamese family with MED.

**Case presentation:**

A standard clinical tests and radiological examination were performed in an 8-year-old Vietnamese girl patient. The clinical examination showed that patient height was under average, with bent lower limbs, limited mobility and dislocation of the joints at both knees. Radiological documentation revealed abnormal cartilage development at the epiphysis of the femur and patella. The patient has a varus deformity of the lower limbs. The patient was diagnosed with autosomal dominant MED using molecular testing in the order of the coding sequences and flanking sequences of five genes: COMP (exons 8–19), MATN3 (exon 2), COL9A2 (exon 3), COL9A3 (exon 3), COL9A1 (exon 8) by Sanger sequencing. A novel heterozygous missense variant (c.572 C > A, p.A191D) in *MATN3* was identified in this family, which were not inherited from parents. The p.A191D was predicted and classified as a pathogenic variant. When the two predicted structures of the wild type and mutant matrilin-3 were compared, the p.A191D substitution caused conformational changes near the substitution site, resulting in deformity of the β-sheet of the single A domain of matrilin- 3.

**Conclusions:**

This is the first Vietnamese MED family attributed to p.A191D matrilin-3 variant, and our clinical, radiological and molecular data suggest that the novel de novo missense variant in *MATN3* contributed to MED.

## Background

Multiple epiphyseal dysplasia (MED) is a skeletal dysplasia of varying severity and is characterized by variable degrees of epiphyseal abnormality primarily involving the hip and knee joints [1–3]. The prevalence of autosomal dominant MED is estimated to be at least 1 in 10,000 newborns [4]. To date, mutation in five different genes have been shown to cause autosomal dominant MED; the genes encoding matrilin-3 (*MATN3*), cartilage oligomeric matrix protein (*COMP*), and the alpha 1–3 chains of type IX collagen (*COL9A1, COL9A2, COL9A3*) [2]. The relative proportions of five genes contributed to MED are different depending on ethnicity. A study of Jackson and his colleagues in the European Skeletal Dysplasia Network found that *MATN3* pathogenic variants accounted for 24%, *COMP* for 66%, *COL9A2* for 8%, and *COL9A3* for 2% [5]. Whereas, the proportion of autosomal dominant MED attributed to MATN3 pathogenic variants was 55%, followed by that to COMP (43%), and COL9A2 (2%) reported in 55 Korean individuals [6].

*MATN3* incorporates coding sequence that comprises eight exons [7]. To date, at least 20 different variants in the *MATN3* have been shown to cause a mild form of MED [8]. All of these missense variants and the polymorphism are found in the single A-domain of matrilin-3, which is encoded by exon 2 of *MATN3* [4]. Matrilin-3 is the third member of a family of four extracellular matrix (ECM) proteins [9]. It consists of a single A-domain, four EGF repeats, and a coiled-coil domain that facilitates oligomerization. It forms either homotetramers or heterotetramers with matrilin-1 [1, 10]. Matrilin − 3/1 isolated from cartilage has been shown to bind with high affinity to both type IX collagen and cartilage oligomeric matrix protein, are believed to act as adaptor proteins in the ECM [1, 11].

In the present study, we used molecular testing that was shortened by targeting various exons that regularly occur variants in five genes cause MED in the order. The current study assessed in the order of the coding sequences and flanking sequences of five genes: *COMP* (exons 8–19), *MATN3* (exon 2), *COL9A2* (exon 3), *COL9A3* (exon 3), *COL9A1* (exon 8) associated with MED by Sanger sequencing, and characterized the associated clinical features. We identified a novel p.A191D matrilin-3 variant associated with MED from a Vietnamese family. Genetic analysis revealed heterozygous for the novel de novo missense variant in *MATN3*. Our genetic studies and clinical evaluation suggest that the novel p.A191D matrilin-3 is a pathogenic variant contributed to MED.

## Case presentation

### Patient information

**Fig. 1:**
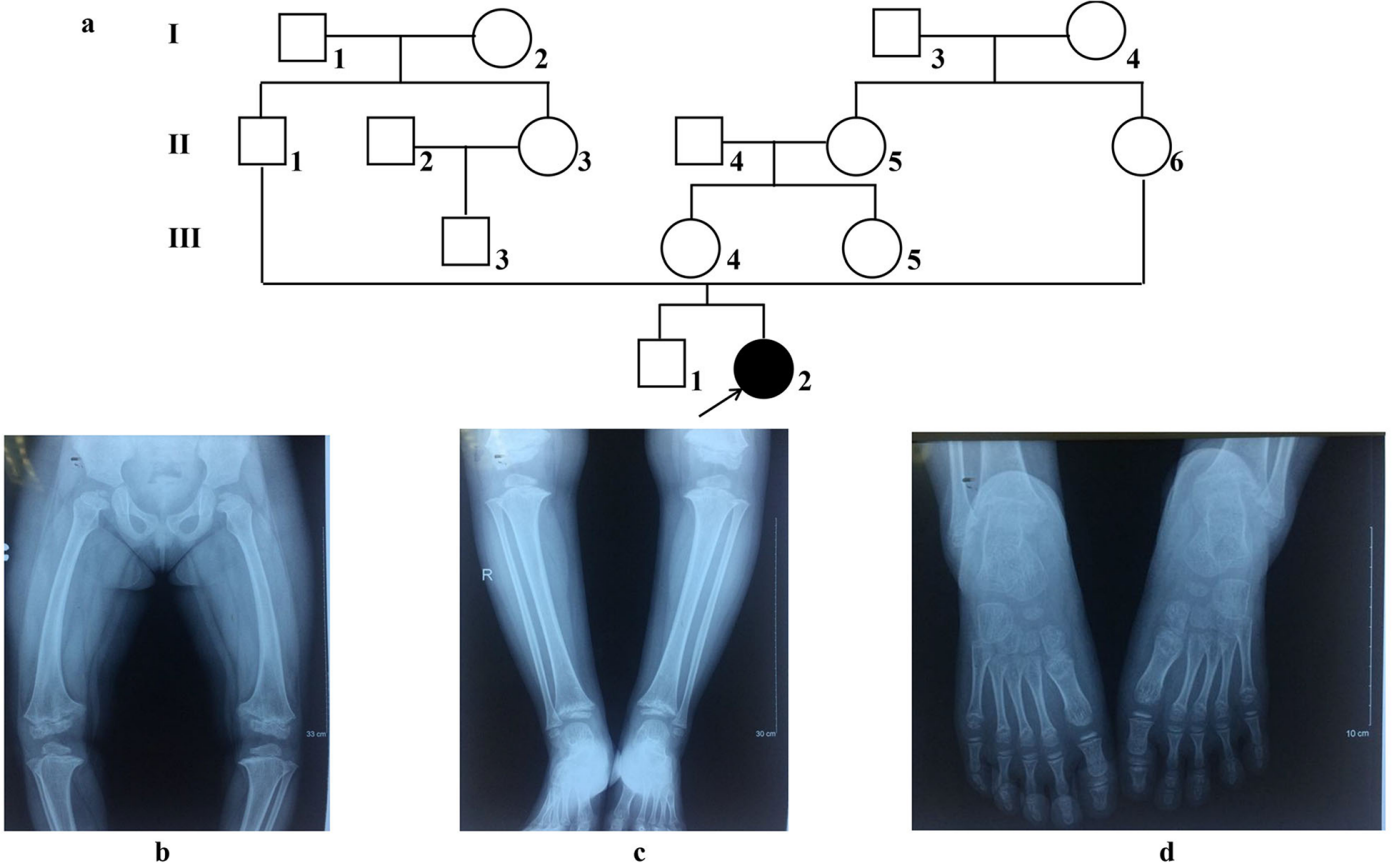
Clinical manifestations of patient. **a** Personal and family history of the Vietnamese family presenting with MED. Squares denote males, circles denote females. **b**, **c**, **d**. Radiographs of the patient. The patient has a varus deformity of the lower limbs. Her limb lengths were lower than average: her femur was 33 cm long (**b**), and her tibia was 30 cm (**c**). The patient had abnormal cartilage development at the epiphysis of the femur and patella. Ossification occurred prematurely at the epiphyseal plates. The metaphysis of the femur and patella was deformed and flatten; the femur condyle was shorten. No deformity was detected in her feet (**d**)

The patient was an 8-year-old girl of two healthy parents. She has short stature of 105 cm height and weight of 40 kg. She has normal intellectual abilities. Her physical examination showed that her height was under average, with bent lower limbs (genu varum), limited mobility and dislocation of the joints at both knees. Personal and family histories were reviewed for each member of the three-generation Vietnamese family (Fig. 1a).

### Past medical history

Back in her prenatal stage, at 22 week old, the sonogram revealed the shortness of the limbs. However, the family still decided to keep the baby. There was no family history of musculoskeletal problems. Her karyotype results were 46, XX, with no abnormalities detected at chromosomal level (data not shown).

As the patient learnt to walk, the bending of the legs became more pronounced. She was admitted to the hospital and was provided with a cast for her legs in 3 months. After removing the cast, her condition was not improved. At the age of 6, the patient received a knee arthrodesis procedure.

### Radiological findings

The patient has a varus deformity of the lower limbs. Her limb lengths were lower than average: her femur was 33 cm long, and her tibia was 30 cm (Fig. 1b, c).

Radiological documentation revealed abnormal development in the femur and patella as ossification of the epiphyseal growth plates occurred irregularly. Tibial metaphysis are deformed and flatten; the femoral heads were short and flat. No deformity was detected in her hands and feet (Fig. 1d).

### Molecular data

The study was approved by the Institute of Biotechnology and Hanoi Medical University Hospital (Ha Noi, Vietnam), and informed consent was obtained from all family members prior to blood sample collection. All family members from a Vietnamese family were recruited at Hanoi Medical University Hospital (Ha Noi, Vietnam) between April and May 2019. Genomic DNA samples were then extracted from the peripheral blood leucocytes of all family members using the Qiagen QIAamp DNA Mini kit (Qiagen Inc., Valencia, CA, USA) according to manufacturer’s protocol. DNA concentration and purity was measured using a NanoDrop™ ND-1000 spectrophotometer (Thermo Fisher Scientific, Inc., Waltham, MA, USA). Genomic DNA samples were preserved at − 20 °C prior to use.

All sequences of exons 8–19 (*COMP*), exon 2 (*MATN3*), intron 3-exon 3 (*COL9A2*), intron 2-exon 3 (*COL9A3*), intron 7-exon 8 (*COL9A1*) were amplified in family members using PCR with Taq DNA polymerase (Thermo Fisher Scientific, Inc.), and specific primers sequences for *COMP*, *MATN3*, *COL9A2* designed by previous studies [1, 7, 12], respectively. We designed new primers for amplification of target regions of *COL9A3* and *COL9A1*. All primer sequences were listed in Supplementary 1. DNA (100 ng) in a 50 μl reaction was amplified. All reagents used for PCR were purchased from Thermo Fisher Scientific, Inc. The amplification included a single 5 min step at 94 °C followed by 40 cycles of 94 °C for 45 s, annealing at temperatures listed (see Table 1) for 45 s and 72 °C for 45 s followed by a final 10 min step at 72 °C. Products were separated using 1% agarose gel electrophoresis and stained with ethidium bromide. DNA products then were purified using a QIAquick PCR Purification kit (Qiagen Inc.) and sequenced in each direction using an ABI3100 Genetic Analyzer (Thermo Fisher Scientific, Inc.). The variant detection was firstly performed for *COMP* and expanded to *MATN3*, *COL9A1*, *COL9A2*, *COL9A3* in order by Variant Reporter™ Software v2.0, Initial License (Thermo Fisher Scientific, Inc.), and analysed using SeqMan (version 2.3; Technelysium Pty, Ltd., South Brisbane, QLD, Australia), and compared against reference sequences obtained from NCBI with the code of NG_007070.1 (*COMP*), NG_008087.1 (*MATN3*), NC_000001.11 (*COL9A2)*, NG_016353.1 (*COL9A3*), NG_011654.1 (*COL9A1*).

**Table 1:** Functional prediction of a novel de novo missense variant in *MATN3*

Chr.	Position	Gene symbol	Transcript variant	Protein variant	Mutation Taster score	Polyphen-2	SIFT	Effect
2	20,205,723	*MATN3*	572C > A	p.A191D	0.999	1.000	0.02	- Damaging - Disease causing (amino acid sequence changed) - Protein features (might be) affected) - Affect protein function

**Fig. 2:**
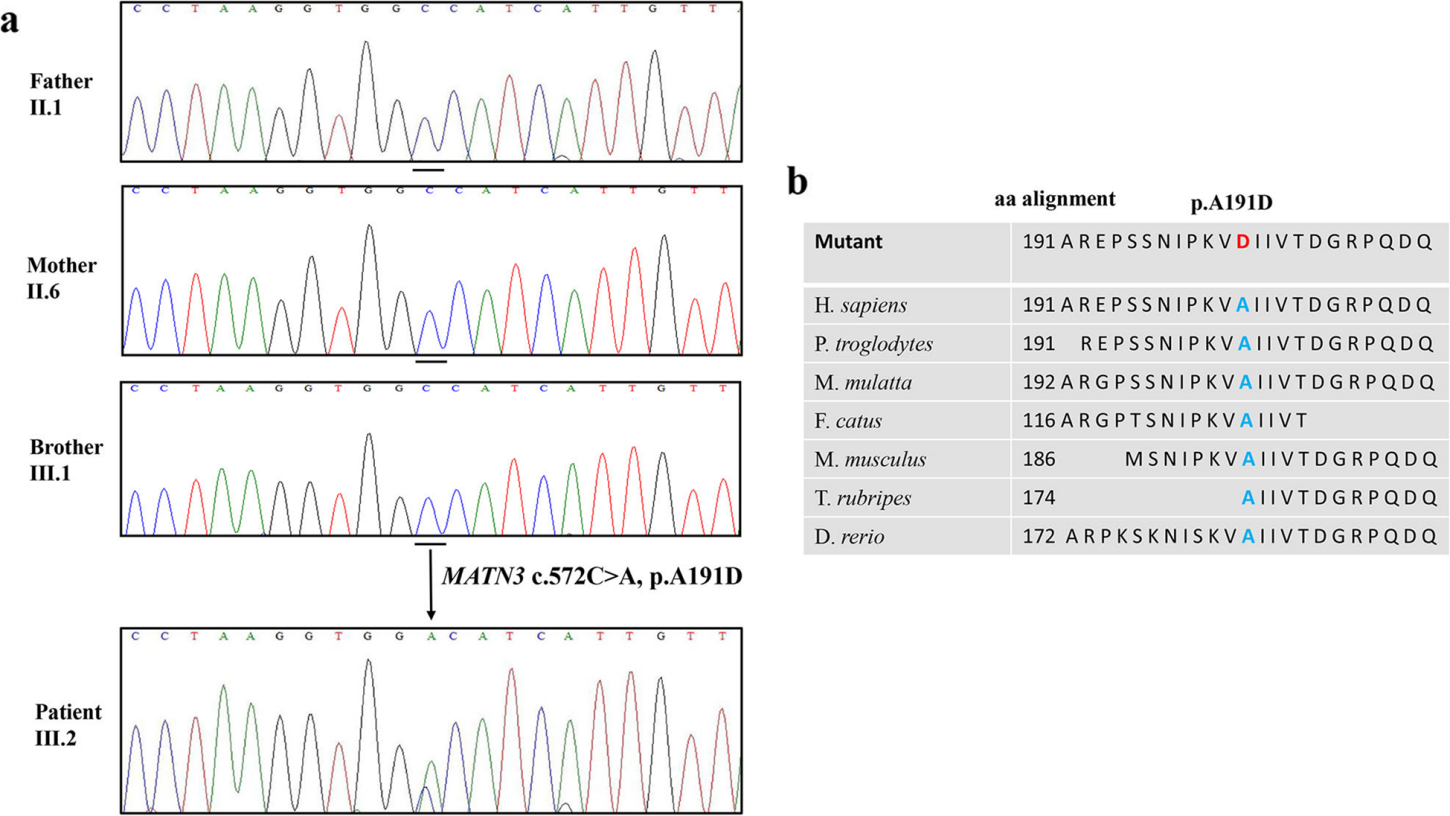
Identification of a novel de novo missense variant in *MATN3* (**a**) The DNA-sequence electropherogram of parents and brothercompared with that of patient. A heterozygousmissense variant (c.572C > A; p.A191D) in exon 2 of the *MATN3* was identified. (**b**) The variant is evolutionarily conserved

The Sanger sequencing revealed a de novo heterozygous variant at cDNA position c.572C > A in the exon 2 of the *MATN3* in the patient, but not in unaffected parent and unaffected brother (Fig. 2a). This missense variant changes the codon for Alanine to Aspartic acid at the protein level (p.A191D). The heterozygous c.572C > A variant in exon 2 of *MATN3* is evolutionarily conserved among species (Fig. 2b). The following criteria were used to determine if the sequence variation was pathogenic variant: the chemical nature of the amino acids substituted, co-segregation with phenotype in family, interspecies amino acid conservation, absence in healthy individuals. Protein damaging structural information of the missense variant (c.572C > A, p.A191D) was predicted in MutationTaster [13], Polyphen-2 [14] and SIFT [15] to be ‘Damaging’ with a score of 0.999, ‘Disease causing’ with a score of 1.000, and ‘Affect protein function’ with a score of 0.02, respectively (Table 1). Based on the standards and guidelines for the interpretation of sequence variants of the American College of Medical Genetics and Genomics and the Association for Molecular Pathology [16], the c.572C > A (p.A191D) variant is classified to be ‘Pathogenic’. In addition, the variant has not been found in any public database, including dbSNP (http://www.ncbi.nlm.nih.gov/projects/SNP/), 1000 genomes (http://www.1000genomes.org/), Human Gene Mutation Database (http://www.hgmd.cf.ac.uk/ac/index.php), Genome Aggregation Consortium (https://gnomad.broadinstitute.org/), Human Genetic Variation Database (http://www.hgvd.genome.med.kyoto-u.ac.jp/), ClinVar (https://www.ncbi.nlm.nih.gov/clinvar/), ESP6500 (http://evs.gs.washington.edu/EVS/). Therefore, the de novo missense variant (c.572C > A, p.A191D) was considered to be novel.

**Fig. 3:**
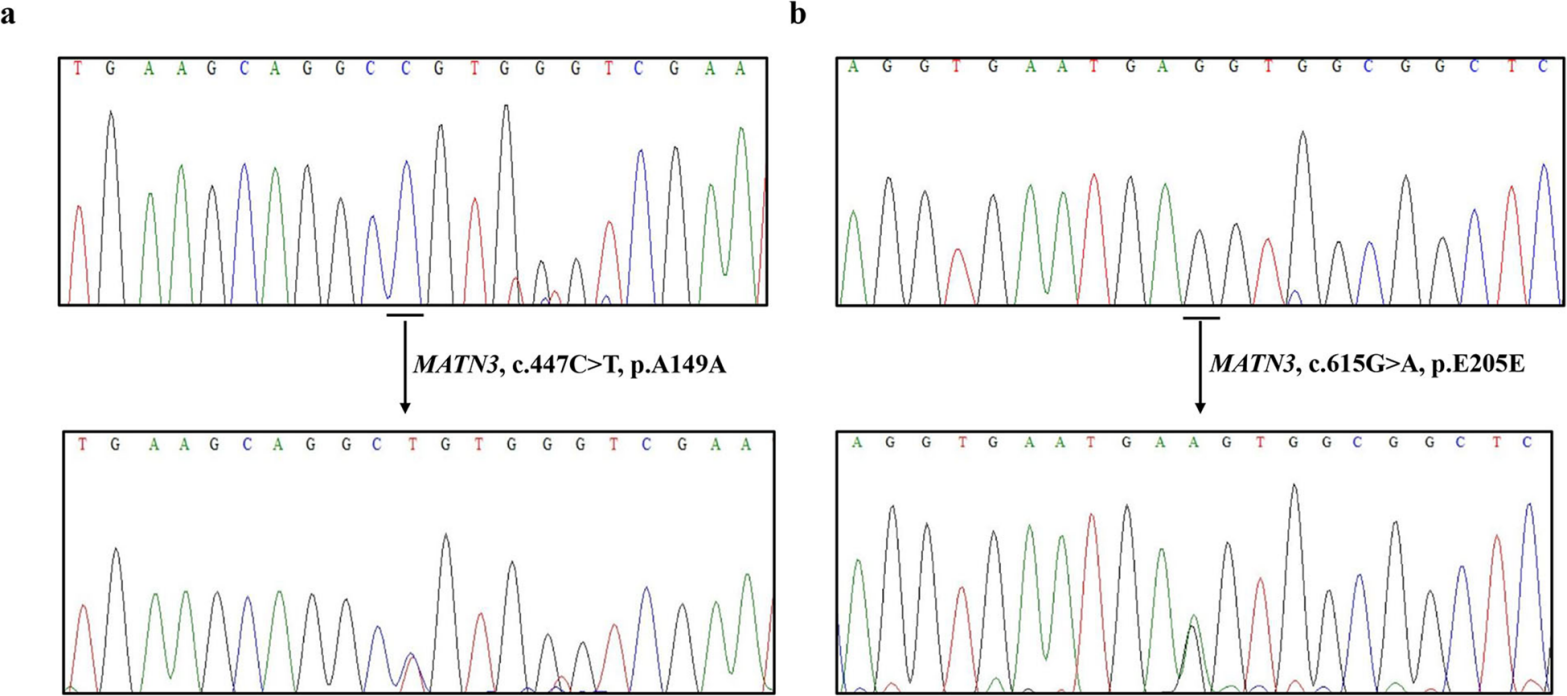
Identification of two known coding-synonymous variants in *MATN3.***a** The DNA-sequence electropherogram of c.447C > T coding-synonymous variant. **b** The DNA-sequence electropherogram of c.615G > A coding-synonymous variant

Moreover, two known coding-synonymous variants: c.447C > T, c.615G > A in exon 2 of *MATN3* were identified in patient and unaffected father, unaffected brother (Fig. 3). These variants are not conserved. The frequencies of these variants are above 40% in several public databases: 1000 Genomes, ExAC, GnomAD_exomes and Vietnamese. Based on the standards and guidelines for the interpretation of sequence variants of the American College of Medical Genetics and Genomics and the Association for Molecular Pathology [16], these variants are classified to be ‘Benign’ for pathogenesis. All variants detected in this family, the variant frequencies in the population and variant classifications were shown in Table 2.

**Table 2:** List of variants in *MATN3* in family

	Variant	Variant classification
**Father II.1**	c.447C > T, p.(=)	Benign
	c.615G > A, p.(=)	Benign
**Mother II. 6**	–	
**Brother III.1**	c.447C > T, p.(=)	Benign
	c.615G > A, p(=)	Benign
**Patient III.2**	c.447C > T, p. (=)	Benign
	c.615G > A, p.(=)	Benign
	c.572C > A, p.A191D	Pathogenic

(−): No variant was detected

**Fig. 4:**
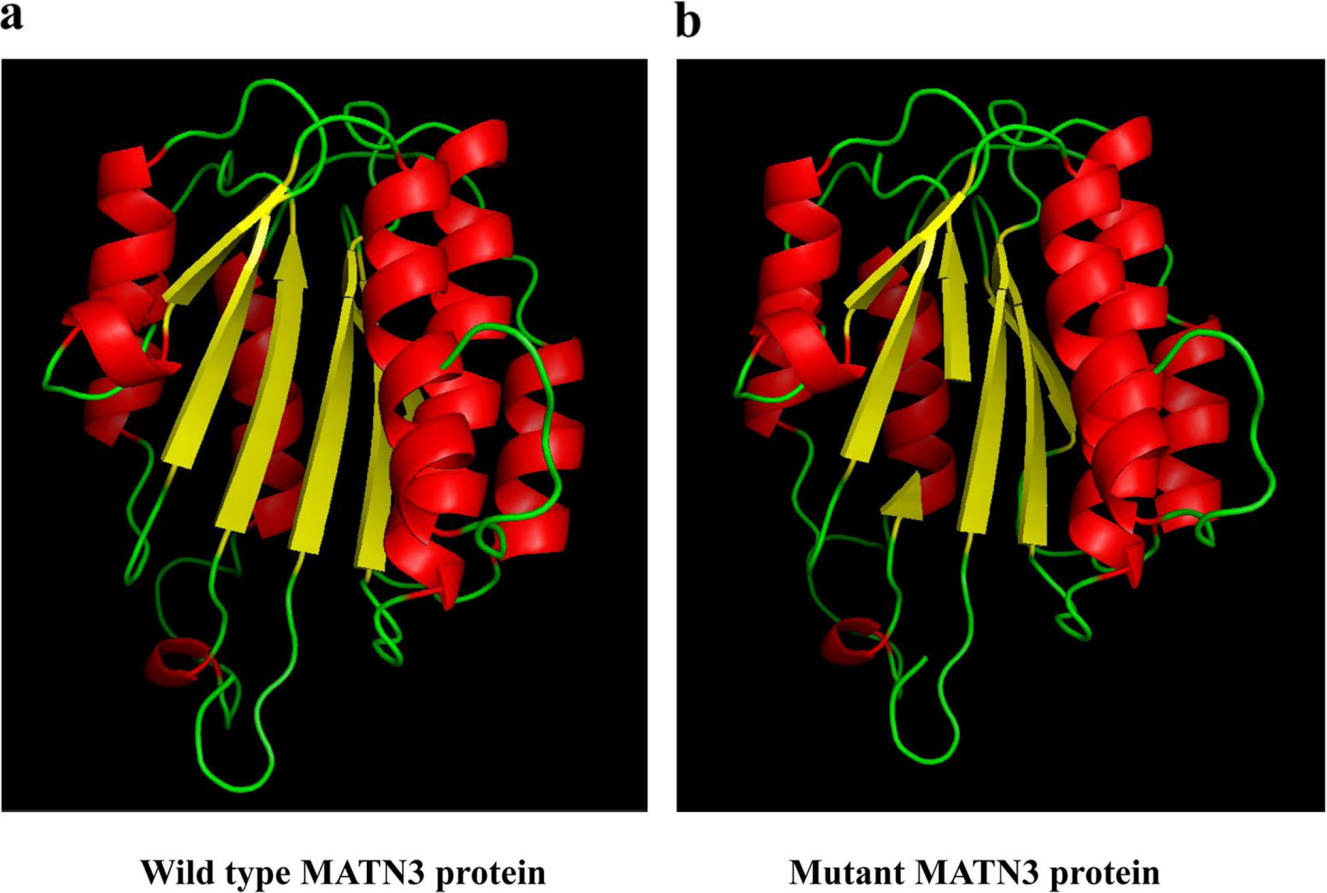
Modeling studies of novel p.A191D variant in MATN3. **a** The model of the tertiary structure of human matrilin-3 (UniProt ID: O15232) as the wild type was constructed by SWISS-MODEL. **b** The model of the tertiary structure of mutated human matrilin-3was built by introducing the p.A191D variant into the wild type model using PyMOL v1.3. The p.A191D substitution caused conformational changes near to the variant site, resulting in losing a part of β-sheet of the single A domain of matrilin-3. β-sheet and α-helix structures of matrilin-3 were shown as yellow and red color, respectively

To understand the consequences of the Alanine to Aspartic acid substitution at amino acid position 191 in matrilin-3 protein, we used the model of the tertiary structure of human matrilin-3 (UniProt ID: O15232) constructed by SWISS-MODEL [17–21] as the wild-type (Fig. 4a) and introduced the p.A191D variant into this model using PyMOL v1.3 (Schrödinger, LLC, New York, USA) to create the mutantmatrilin-3 protein (Fig. 4b). When the two predicted structures of the wild type and the mutatedmatrilin-3 were compared, the p.A191D substitution caused conformational changes near the mutation site, resulting in deformity of the β-sheet of the single A domain of matrilin- 3.

## Discussion and conclusion

In the current study, we shortened molecular testing of the patient by targeting various exons that regularly occur mutations in five genes cause MED in the order. We identified a novel de novo missense variant (c.572C > A, p.A191D) and two known coding-synonymous variants (c.447C > T, p.(=); c.615G > A, p.(=)) in *MATN3* in the patient. The novel c.572C > A, p.A191D variant was predicted by three predictors as ‘Disease causing’ with autosomal dominant MED, suggesting that it is pathogenic variant. Whereas two coding-synonymous variants (c.447C > T, p.(=); c.615G > A, p.(=)) with allele frequencies above 40% in the general population are classified as benign variants. These data therefore extend the range of *MATN3* variants identified to date. Several missense variants in *MATN3* have been previously reported to be associated with MED, including p.R121W, p.V194D [22], p.T120M, p.E134K, p.I192N, and p.A219D [23], p.T120M, p.R121W, p.L146R, p.G159G, p.I192T, p.R209P, p.V220A [6]. All the MED variants in *MATN3* are missense variants found in exon 2 [23].

The severity of pathogenic variants can modify the severity of MED signs and symptoms [4, 23, 24]. MED symptom resulting from *MATN3* pathogenic variants is characterized by significant involvement in knee abnormalities that are similar to those in individuals with a *COL9A2* pathogenic variant; the hip abnormalities are more severe (although not as severe as those in individuals with a *COMP* pathogenic variant) [4, 25]. However, more intra- and interfamilial variability is evident in MED caused by *MATN3* pathogenic variants [4]. In this study, our patient carried p.A191D variant had under average height, with bent lower limbs, limited mobility and dislocation of the joints at both knees. Radiological analysis showed that she had abnormal development in the femur and patella because ossification of her epiphyseal growth plates occurred irregularly. Various missense variants in *MATN3* that is near to the p.A191D variant in our patient were reported with diverse MED symptoms. Kim and colleagues identified a patient carrying a p.I192T variant located in β-sheet of the single A domain of matrilin-3. The patient in that study was 8-year-old boy who had MED symptom of gait abnormality, but he had normal height. Radiographic features of his hips indicated that there was a dense, crescent shape of the femoral heads with fragmentation on the left side mimics Legg-Calve-Perthes disease [6]. In addition, there was another p.V194D variant that is next to the mutation in our patient was identified by Chapman and his colleagues [22]. That patient presented at the age of 10 years with knee pain after exercise, genu valga and normal stature. Radiographs of his skeleton revealed that he had normal spines and hands but predominant involvement of the epiphyses of hips, knees and ankles [22]. Therefore, these supports previous observations that the severity of MED signs and symptoms can vary among affected patients due to severity of the pathogenic variants even with very close variants [23, 24].

The p.A191D variant in matrilin-3 is located within the central β-sheet of the single A domain of matrilin-3. The change of Alanine to Aspatic acid at position 191 caused conformational changes near to the substitution site, resulting in malformation of β-sheet of the single A domain of matrilin-3. The consequences of various missense variants in the β-strands of the A domain of matrilin-3 protein had been reported. The previous study demonstrated that various missense variants in the β-strands of the A domain of matrilin-3 protein delayed folding, prevented correct intra-molecular disulfide bond formation, and results in the retention of mutated matrilin-3 in the rough endoplasmic reticulum both in vitro [1, 26] and in vivo [27, 28]. Leighton and his colleagues indicated that the expression of p.V194D pathogenic variant caused endoplasmic reticulum stress and an unfolded protein response in an MED mouse model [27]. In addition, matrilins have been found in collagen-dependent and collagen-independent filament networks within the tissues in which they are expressed and may perform analogous functions in these different tissues [4, 29, 30]. Moreover, matrilin-3 has been shown to interact with high affinity to both cartilage oligomeric matrix protein and type IX collagen through A domain, and act as adaptor proteins in the ECM [10, 29–31]. Therefore, the distortion of β-sheet of the single A domain of matrilin-3 caused by p.A191D = variant might affect to the adaptor protein function of matrilin-3, and might lead to low binding affinity to cartilage oligomeric matrix protein and type IX collagen protein.

In summary, a novel de novo missense variant c.572C > A (p.A191D) in exon 2 of the *MATN3* with evolving clinical and radiological features of MED was identified in a Vietnamese family. These results may not only expand the reported variant spectrum of *MATN3*, but may also provide useful information to aid clinicians to confirm the diagnosis of MED. More genetic studies are required to determine the frequency of this variant and this diagnostic credibility.

## Supplementary information

**Additional file 1.** Primer sequences for amplification.

**Additional file 2.** The frequencies of two known variants in public database.

## Data Availability

The datasets used and/or analysed during the current study are available from the corresponding author on reasonable request.

## Abbreviations

MED: Multiple epiphyseal dysplasia
*MATN3*: Gene encoding matrilin-3
*COMP*: Gene encoding cartilage oligomeric matrix protein
*COL9A1*, *COL9A2*, *COL9A3*: Gene encoding the alpha 1–3 chains of type IX
ECM: Extracellular matrix
SNP: Single nucleotide polymorphism
