# Supplementary material for: A novel p.A191D matrilin-3 variant in a Vietnamese family with multiple epiphyseal dysplasia: a case report
Source: BMC Musculoskelet Disord. 2020 Apr 7;21:216. doi: 10.1186/s12891-020-03222-4 (PMC7140548; doi:10.1186/s12891-020-03222-4)
Supplement: Supplementary file 1 — Additional file 1. Primer sequences for amplification. [file 12891_2020_3222_MOESM1_ESM.docx]

**Supplementary 2. The frequencies of two known variants in *MATN3* in public database**

| **Variant** | **Variant classification** | **Variant frequency in public database** | | | |
| --- | --- | --- | --- | --- | --- |
|  |  | **1000Genomes** | **ExAC** | **GnomAD_exomes** | **Vietnamese** |
| c.447C>T,  p.(=) | Benign | G=47.524% | A=47.5389% | A=47.4854% | G=40.3909% |
| c.615G>A,  p.(=) | Benign | C=47.5639% | T=48.3708% | T=47.3495% | C=41.0596% |
