# Supplementary material for: A novel p.A191D matrilin-3 variant in a Vietnamese family with multiple epiphyseal dysplasia: a case report
Source: BMC Musculoskelet Disord. 2020 Apr 7;21:216. doi: 10.1186/s12891-020-03222-4 (PMC7140548; doi:10.1186/s12891-020-03222-4)
Supplement: Supplementary file 2 — Additional file 2. The frequencies of two known variants in public database. [file 12891_2020_3222_MOESM2_ESM.docx]

**Supplementary 1. Primer sequences for amplification**

| **Gene** | **Target** |  | **Primer sequences (5’-3’)** | **Annealing (ºC)** | **Size** | **References** |
| --- | --- | --- | --- | --- | --- | --- |
| *COMP* | Exons 8-9 | 8F | TTGAGGCGGGGTTGGGTG | 59 | 413 | Kennedy et al., 2005 |
|  |  | 9R | ACCGTGCCGAGCCGTAGAT |  |  |  |
|  | Exon 10 | 10F | AGGAGTGTGACCTTTGCCTTCT | 59 | 334 |  |
|  |  | 10R | CTAGTCCAGCTTACCCCATCC |  |  |  |
|  | Exons 11-12 | 11F | GAAGTCATTCTGGCCTGGTC | 59 | 518 |  |
|  |  | 12R | AGCGTTTTGTCA AAGGCTACC |  |  |  |
|  | Exon 13 | 13F | CGGGTAGCCTTTGACAAA ACG | 57 | 331 |  |
|  |  | 13R | GCCCGCCCACCGTAG AC |  |  |  |
|  | Exons 14-15 | 14F | GGCGGGCCCTGACTT TAG | 59 | 546 |  |
|  |  | 15R | ATA ACCCCGCCCCTCTGT |  |  |  |
|  | Exon 16 | 16F | GTTCTGGGT GCCAGGTTC | 59 | 335 |  |
|  |  | 16R | AAGGGTTTTACGGAGGGTCAT |  |  |  |
|  | Exon 17 | 17F | TGCTCCCAACTGTCTCTCCA | 59 | 312 |  |
|  |  | 17R | ACCTGGGCCTGTGTGTCC |  |  |  |
|  | Exons 18-19 | 18F | TCTGAGAGGGAAGGGTCTGG | 59 | 443 |  |
|  |  | 19R | CCCTTCTCACTTCCCCCTCA |  |  |  |
| *MATN3* | Exon 2 | e.2F | TGCAAGAGCAGACCCTTGGAC | 59 | 561 | Cotterill et al., 2005 |
|  |  | e.2R | ACAGAAGGTTTCCTGGAATCT |  |  |  |
| *COL9A2* | Introns 3-exon3 | intr.3F | CAATGGGCCCCCTGGAAAAGC | 56 | 176 | Muragaki et al., 1996 |
|  |  | intr.3R | CAATCCCGGGCTTCCCGTCTG |  |  |  |
| *COL9A3* | Introns 2-exon 3 | Int.2F | TGCCCTCTAGGTAGGGATCG | 55 | 251 |  |
|  |  | e.3R | TTCCCCCTTTCTCTCCAGACTG |  |  |  |
| *COL9A1* | Introns 7-Exon 8 | Int.7F | GCAGAGGTGAGAACCAGTG | 55 | 391 |  |
|  |  | e.8R | ATAGGAAAGGGCAGGACTGG |  |  |  |
